# Paradoxical Regulation of Human FGF21 by Both Fasting and Feeding Signals: Is FGF21 a Nutritional Adaptation Factor?

**DOI:** 10.1371/journal.pone.0022976

**Published:** 2011-08-01

**Authors:** Takashi Uebanso, Yutaka Taketani, Hironori Yamamoto, Kikuko Amo, Hirokazu Ominami, Hidekazu Arai, Yuichiro Takei, Masashi Masuda, Ayako Tanimura, Nagakatsu Harada, Hisami Yamanaka-Okumura, Eiji Takeda

**Affiliations:** 1 Department of Clinical Nutrition, Institute of Health Biosciences, University of Tokushima Graduate School, Tokushima, Japan; 2 Department of Metabolic Disorder, Diabetes Research Center, Research Institute, National Center for Global Health and Medicine, Tokyo, Japan; 3 Laboratory of Clinical Nutrition Management, School of Food and Nutritional Sciences, The University of Shizuoka, Shizuoka, Japan; 4 Department of Nutrition and Metabolism, Institute of Health Biosciences, University of Tokushima Graduate School, Tokushima, Japan; The University of Hong Kong, Hong Kong

## Abstract

Fibroblast growth factor 21 (FGF21) has recently emerged as a metabolic hormone involved in regulating glucose and lipid metabolism in mouse, but the regulatory mechanisms and actions of FGF21 in humans remain unclear. Here we have investigated the regulatory mechanisms of the human *FGF21* gene at the transcriptional level. A deletion study of the human *FGF21* promoter (−1672 to +230 bp) revealed two fasting signals, including peroxisome proliferator-activated receptor α (PPARα) and glucagon signals, that independently induced human *FGF21* gene transcription in mouse primary hepatocytes. In addition, two feeding signals, glucose and xylitol, also dose-dependently induced human *FGF21* gene transcription and mRNA expression in both human HepG2 cells and mouse primary hepatocytes. FGF21 protein expression and secretion were also induced by high glucose stimulation. The human FGF21 promoter (−1672 to +230 bp) was found to have a carbohydrate-responsive element at −380 to −366 bp, which is distinct from the PPAR response element (PPRE). Knock-down of the carbohydrate response element binding protein by RNAi diminished glucose-induced human *FGF21* transcription. Moreover, we found that a region from −555 to −443 bp of the human *FGF21* promoter region exerts an important role in the activation of basic transcription. In conclusion, human *FGF21* gene expression is paradoxically and independently regulated by both fasting and feeding signals. These regulatory mechanisms suggest that human FGF21 is increased with nutritional crisis, including starvation and overfeeding.

## Introduction

Fibroblast growth factor 21 (FGF21), a newly identified member of the FGF19 subfamily, has recently emerged as a metabolic hormone involved in the regulation of glucose and lipid metabolism in mice [1]–[8]. In particular, FGF21 is induced in the liver by fasting and regulates gluconeogenesis, fatty acid oxidation, ketogenesis and torpor in mice [2], [4], [7]. In addition, FGF21 stimulates glucose uptake in adipocytes and protects from diet-induced obesity and metabolic disorders in obese or diabetic animals given FGF21 and in FGF21 transgenic mice [2], [3], [5], [8].

Relatively little is known about the regulation of FGF21 in humans as compared to mice. In a human study, fasting for 7 but not 2 days was found to induce FGF21 [9]. On the other hand, some human studies have shown that serum FGF21 levels are increased in both obese individuals and patients with type 2 diabetes, which suggest that hyperglycemia and/or hyperinsulinemia can regulate *FGF21* gene expression in humans [10], [11]. In the present study, we show that human *FGF21* gene expression was regulated by glucose via ChREBP. In addition, two fasting signals, PPAR α and glucagon also increased human *FGF21* gene transcription. Thus, this study helps to clarify the paradoxical regulation of human *FGF21* by both fasting and overfeeding, and provides clues to understand the potential roles of FGF21 in humans.

## Materials and Methods

### Construction of plasmid vectors

PCR was performed with an Expand High-Fidelity PCR system (Roche) and the appropriate primers (Invitrogen). Luciferase gene constructs containing −1672 to +230 bp of the human *FGF21* promoter (pFGF21-1.6k) reporter vector and a series of 5′-deletion mutant vectors (pFGF21-1k, pFGF21-555, pFGF21-443, pFGF21-289 and pFGF21+11) were prepared from human genomic DNA and pGL4.12 vectors (Promega). The sequence of mouse short hairpin carbohydrate response element binding protein (ChREBP) reported by Dentin et al. was used as a suitable sequence for RNAi targeting [12]. Double-stranded oligonucleotides encoding ChREBP or LacZ were synthesized, annealed and cloned into a pEnter/U6 entry vector (Invitrogen).

### Cell culture

Primary hepatocytes were isolated from normal male ddy mice (20–25 g; Japan SLC) by using a collagenase perfusion method as described previously [13]. The University of Tokushima Animal Use Committee approved the study, and mice were maintained according to the National Institutes of Health guidelines for care and use of laboratory animals (approval ID, 08052). Hepatocyte suspensions were plated on 35-mm plastic dishes in a final volume of 2 ml of William's E medium (Sigma) supplemented with 1 nmol of insulin (Sigma), 1 nmol of dexamethasone (Sigma), 10% (v/v) fetal bovine serum (Invitrogen) and 1% (v/v) penicillin/streptomycin (Sigma). After 6 h of attachment, the medium was removed and fresh, serum-free medium was added. After 12 h in culture, the cells were stimulated for the indicated time with medium containing glucose, xylitol or mannitol.

Human hepatoma HepG2 cells were cultured in Dulbecco's modified Eagle's medium (DMEM; Sigma) containing 10% (v/v) fetal bovine serum, 50 IU/ml penicillin, and 50 µg/ml streptomycin. After 12 h in culture with serum-free DMEM, the cells were stimulated with medium containing glucose for the indicated time.

### Transfections and luciferase assays

Transfection studies were carried out in precultured mouse primary hepatocytes or HepG2 cells. The indicated amount of each expression plasmid was transfected simultaneously with a luciferase reporter plasmid (0.5–1.0 µg) and pCMV-β-galactosidase (0.25–0.5 µg, Invitorgen) by using Lipofectamin2000 (Invitrogen). The total amount of DNA in each transfection was adjusted to 1.5 µg/well with pCMV. After 12 h, the cells were cultured with medium containing glucagon, Forskolin, Wy-14643, glucose, xylitol or mannitol for 6 h, and the amount of luciferase activity in transfectants was measured and normalized to the amount of β-galactosidase activity as measured by a standard kit (Promega). Short hairpin ChREBP and the LacZ expression vector (0.5 µg) were transfected 48 h prior to starting the luciferase assay. Transfection efficiency was 48±5% for primary hepatocytes and 52±8% for HepG2 cells. There was no significant difference in the transfection efficiency between the cells.

### RNA preparation and quantitative RT-PCR

Extraction of total RNA, cDNA synthesis and real-time PCR analysis were performed as described previously [13]. The following primers were used for quantitative PCR. β-actin (human): forward, 5′-GGCACCACACCTTCTACAATGAGC-3′; and reverse, 5′-AGCCTGGATAGCAACGTACATGGC-3′. β-actin (mouse): forward, 5′-CTGACCCT GAAGTACCCCATTGAACA-3′; and reverse, 5′-CTGGGGTGTTGAAGGTCTCAAACATG-3′. FGF21 (human): forward, 5′-GGGAGTCAAGACATCCAGGT-3′; and reverse, 5′-GGCTTCGGACTGGTAAACAT-3′. FGF21 (mouse): forward, 5′-CTACCAAGCATA CCCCATCC-3′; and reverse, 5′-GCCTACCACTGTTCCATCCT-3′. *L-pk*: forward, 5′-CTGCCTTCTGGATATCGACT-3′; and reverse, 5′-GAGTCGTGCAATGTTCATCC-3′. *Fasn*: forward, 5′-CTGCAGAGAAGCGAGCATAC-3′; and reverse, 5′-CTTCTGCC AGTGAGTTGAGG-3′. The relative abundance of each target transcript was calculated by normalization to the amount of product amplified from constitutively expressed β-actin mRNA.

### Protein extraction, immunoblot analysis, and ELISA

The cultured hepatocytes and HepG2 cells were lysed by buffer A (10 mM HEPES-KOH [pH 7.8], 0.1 mM EDTA, 5 mM KCl, 0.1% NP40) containing protease inhibitor. After centrifugation at 13,200× g for 5 min at 4°C, the supernatant was collected as a cytosol extract. The pellet was suspended by buffer C (50 mM HEPES-KOH [pH 7.8], 0.1 mM EDTA, 420 mM KCl, 5 mM MgCl_2_, 20% glycerol) containing protease inhibitor. After centrifugation at 13,200× g for 10 min at 4°C, the supernatant was collected as a nuclear extract. Immunoblotting were performed with antibodies against ChREBP (Novus Biologicals), FGF21 (Abnova), β-actin (Sigma) and LaminB (Santa Cruz Biotechnology). Isolated mouse primary hepatocytes were plated in 6-well plate and stimulate indicated dose of glucose. The cultured media was subjected to analysis for FGF21 concentration with FGF21 ELISA kit according to the manufacturer's instructions (R & D systems).

### Electrophoretic mobility shift assays

Electrophoretic mobility shift assays were performed as previously described [14]. In brief, double-stranded oligonucleotide probes for the human FGF21 carbohydrate response element (ChoRE) were generated, annealed and labeled with [γ^32^-P]ATP. The labeled probes were incubated with hepatic nuclear extracts and 2 µg of poly (dI-dC) in binding buffer. The DNA-protein complexes were resolved on a 5% polyacrylamide gel at 150 V for 90 min. The gels were dried and visualized by bioimage analyzer (FLA9000, Fuji Photo Film).

### Statistical analyses

All values are expressed as mean ± SE. The significance of differences was assessed between two groups by using unpaired two-tailed t-tests, and among more than two groups by using ANOVA or the Kruskal-Wallis test. When a significant difference was found by ANOVA or the Kruskal-Wallis test, post hoc analyses were performed by using the Tukey-Kramer protected least significant difference test. Concentration-dependent effects were found by using regression analysis. Differences were considered significant at p<0.05. Statistical analyses were performed by using StatView (version 5.0-J for Windows, SAS Institute, Inc., Cary, NC).

## Results

### Glucose and xylitol induces FGF21 gene expression

Increases in serum FGF21 have been reported in obese and/or impaired glucose tolerance patients; however, it is unclear whether glucose can activate human *FGF21* gene expression. Excess glucose flux promotes the formation of ATP and xylulose-5-phosphate (Xu-5-P) by glycolysis and the hexose monophosphate shunt pathway. We previously showed that glucagon, which increases the AMP/ATP ratio, stimulates *FGF21* gene expression in rat primary hepatocytes [13]. In this study, we focused on Xu-5-P, which activates ChREBP via phosphatase 2A (PP2A) and promotes its nuclear localization and DNA binding. *Lpk* and *Fasn* are well-known to be glucose-response genes targeted by ChREBP [15]. We therefore used *Lpk* and *Fasn* as positive controls in our experiments.

**Figure 1:**
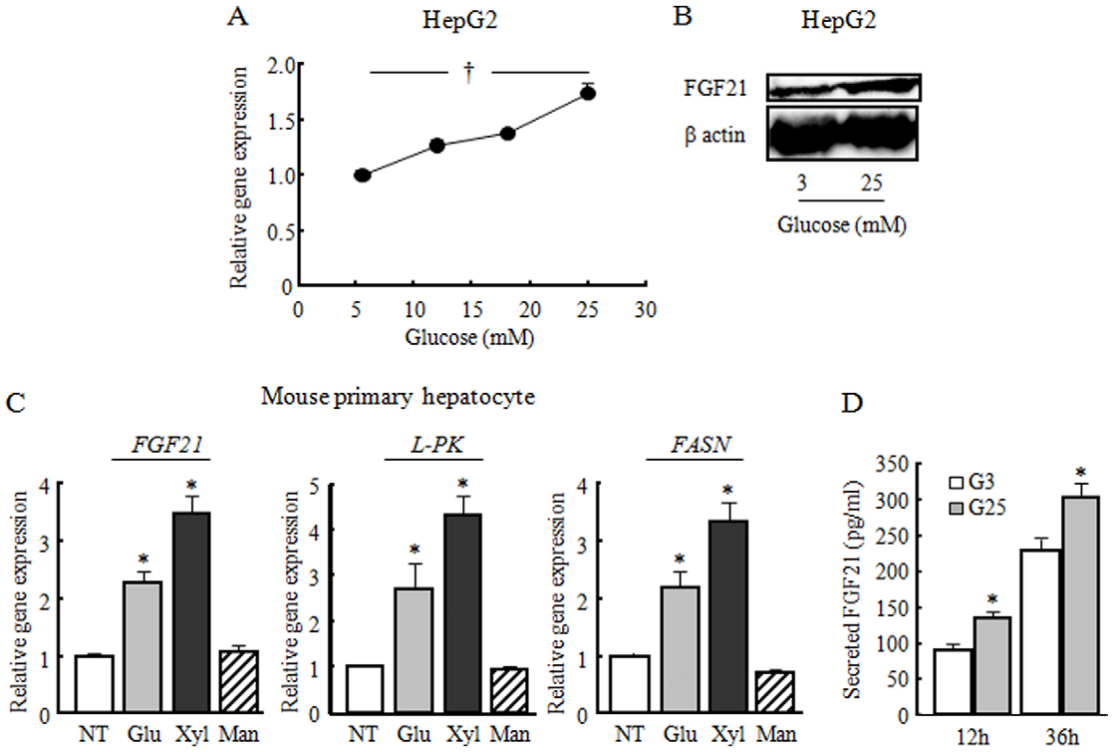
Glucose and xylitol induced FGF21 gene expression in HepG2 and mouse primary hepatocytes. A: Changes in FGF21 gene expression stimulated by the indicated dose of glucose for 6 h in HepG2. B: Changes in FGF21 protein abundance in HepG2 cells stimulated by indicated dose of glucose for 24 h. C: Changes in *FGF21*, *L-pk*, and *Fasn* gene expression stimulated by 10 mM glucose (Glu), xylitol (Xyl) or mannitol (Man) for 6 h in mouse primary hepatocytes. The relative mRNA amount for each gene treated with 5 mM of glucose (A), or NT (non-treatment) (C) was set as 1.0. D: Changes in FGF21 concentration in the cultured media of mouse primary hepatocytes stimulated by 3 mM glucose (G3) or 25 mM glucose (G25) for indicated time. Data represent mean ± SE (n = 3). †: Concentration-dependent effects were observed by regression analysis, p<0.05. *: p<0.05 as compared to NT, mannitol and G3 stimulation.

Glucose dose-dependently induced *FGF21* gene and protein expression in HepG2 cells (Fig. 1A, B). This induction was also observed in mouse primary hepatocytes stimulated by glucose and xylitol, which are metabolized to Xu-5-p (Fig. 1C). Glucose stimulation also increased secretion of FGF21 into the cultured media (Fig. 1D). Glucose and xylitol also induced *Lpk* and *Fasn* mRNA expression (Fig. 1C). In contrast, the same dose of mannitol could not stimulate this gene expression despite having the same osmolarity (Fig. 1C). These results indicate that xylitol can also induce glucose-responsive genes, as reported in an earlier study [16]; therefore, xylitol was used for the subsequent experiments in addition to glucose.

### Both fasting and feeding signals induce human FGF21 promoter activity

**Figure 2:**
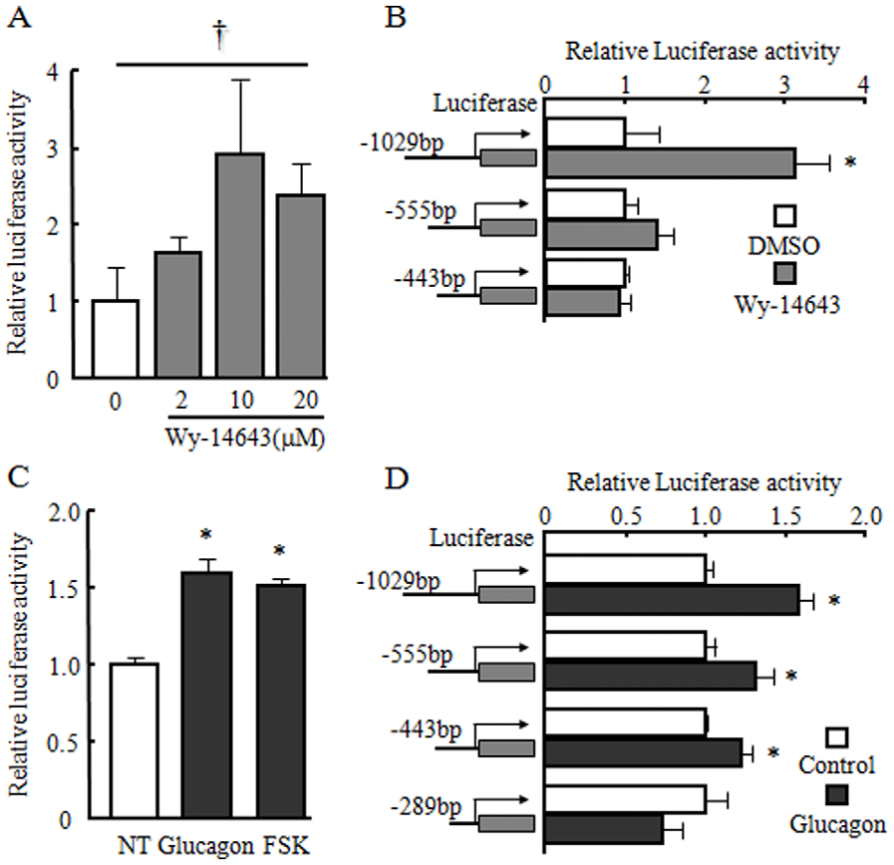
Wy-14643, glucagon and forskolin induced FGF21 gene transcription in mouse primary hepatocytes. A: Changes in relative luciferase activity of a pFGF21-1.6k reporter vector containing the −1672 to +230 bp region of the human *FGF21* promoter simulated by indicated dose of wy-14643 for 6 h in mouse primary hepatocytes. B: Schematic representation of 5′-deletion mutants of the human *FGF21* promoter and changes in relative luciferase activity after stimulation with or without 10 µM wy-14643 for 6 h. C: Changes in relative luciferase activity of the pFGF21-1.6k reporter vector stimulated by 10^−7^ M glucagon or 10^−6^ M forskolin (FSK) for 6 h in mouse primary hepatocytes. D: Schematic representation of 5′-deletion mutants of the human *FGF21* promoter and changes in relative luciferase activity after stimulation with or without 10^−7^ M glucagon. The relative luciferase activity treated with DMSO (A), or NT (non-treatment) (C) was set as 1.0. For deletion experiments (B and D), the relative luciferase activity of each deletion constructs treated with DMSO (B) or control (D) was set as 1.0. Data represent mean ± SE (n = 3). †: Concentration-dependent effects were observed by regression analysis, p<0.05. *: p<0.05 as compared to DMSO or NT.

To investigate further the mechanisms underlying the induction of human *FGF21* gene expression by glucose, we constructed luciferase reporter vectors containing a series of 5′-deletions in the human *FGF21* promoter. It has been reported that the human *FGF21* promoter has a putative peroxisome proliferator-activated receptor (PPAR) response element (PPRE) in the −696 to −685 bp region [17]. We confirmed that wy-14643, a PPARα agonist, dose-dependently increased pFGF21-1.6k promoter activity in mouse primary hepatocytes (Fig. 2A). This response was seen in pFGF21-1k but not in the pFGF21-555 and pFGF21-289 deletion mutant vectors (Fig. 2B). We also investigated the effect of another fasting signal, glucagon-PKA, on human *FGF21* transcriptional activity. The PKA activators glucagon and forskolin significantly increased pFGF21-1.6k promoter activity (Fig. 2C). This response was seen in pFGF21-555 and pFGF21-443 but not in pFGF21-289 deletion mutant vectors (Fig. 2D).

**Figure 3:**
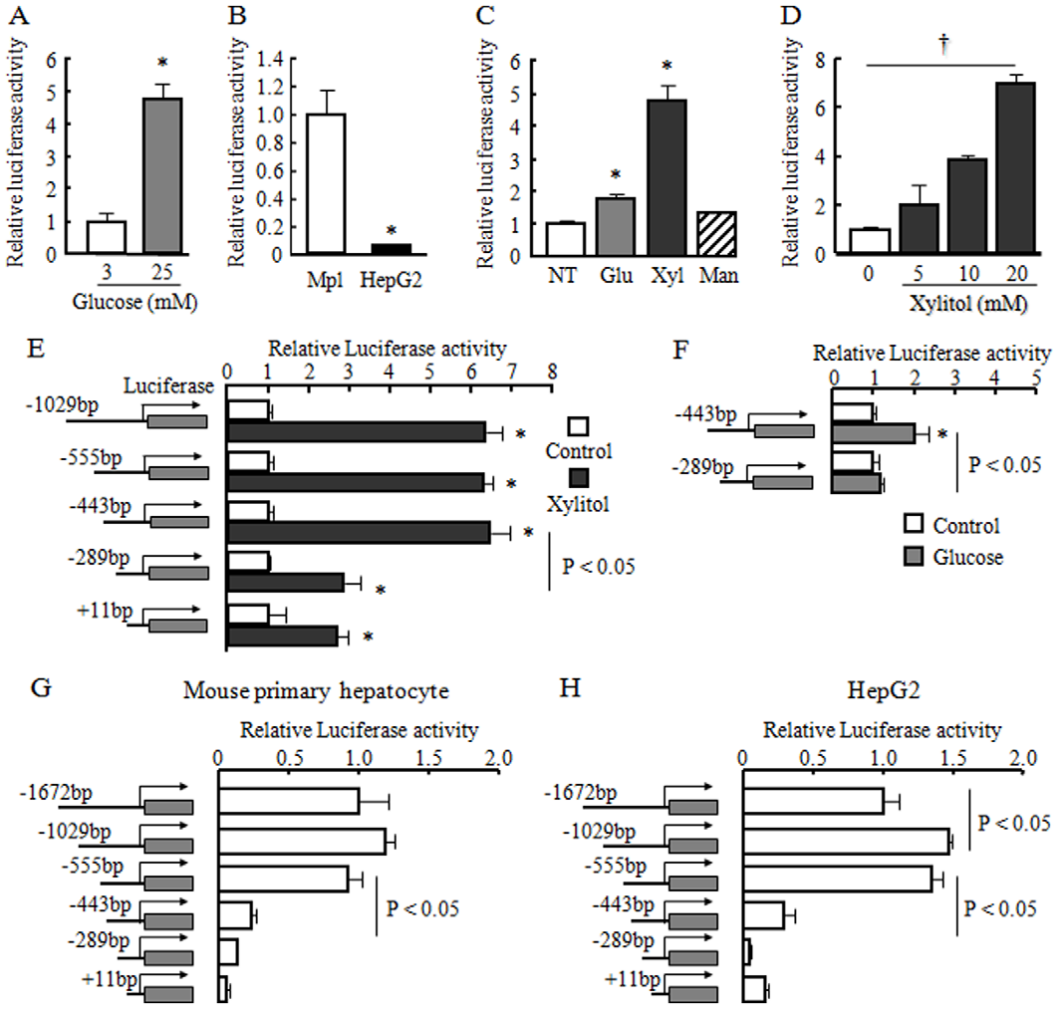
Glucose and xylitol induced human FGF21 gene transcription in mouse primary hepatocytes and HepG2 cells. A: Changes in relative luciferase activity of the pFGF21-1.6k reporter vector stimulated by indicated dose of glucose for 24 h in HepG2 cells. B: Basal luciferase activity of the pFGF21-1.6k reporter vector in mouse primary hepatocytes (Mpl) and HepG2 cells. C: Changes in relative luciferase activity of the pFGF21-1.6k reporter vector stimulated by 10 mM glucose (Glu), xylitol (Xyl) or mannitol (Man) for 6 h in mouse primary hepatocytes. D: Changes in relative luciferase activity of the pFGF21-1.6k reporter vector stimulated by the indicated dose of xylitol for 6 h in mouse primary hepatocytes. E, F: Schematic representation of 5′-deletion mutants of the human FGF21 promoter and changes in relative luciferase activity after stimulation with or without 20 mM xylitol (E) or 20 mM glucose (F). G, H: Basal luciferase activities of 5′-deletion mutants of the human *FGF21* promoter in mouse primary hepatocytes (G) and HepG2 cells (H). The relative luciferase activity for C through F was expressed as described in the Figure 2. For basal luciferase activity analysis (G and H), the relative luciferase activity of -1672 bp was set as 1.0. Data represent mean ± SE (n = 3). †: Concentration-dependent effects were observed by regression analysis, p<0.05. *: p<0.05 as compared to the control.

Next, we investigated the effect of feeding signals, including glucose and xylitol, on human *FGF21* transcription. Glucose increased pFGF21-1.6k promoter activity in HepG2 cells (Fig. 3A). However, the basal reporter activity was 15-fold lower in HepG2 cells than in mouse primary hepatocytes (Fig. 3B). Glucose and xylitol, but not mannitol, stimulated pFGF21-1.6k promoter activity in mouse primary hepatocytes (Fig. 3C, D). We then tested which region of the promoter is important for glucose-dependent induction of the human *FGF21* gene. Xylitol induced approximately 7-fold activation in the pFGF21-1.6k, pFGF21-1k, pFGF21-555 and pFGF21-443 deletion mutant vectors, but induction was reduced to 3-fold in the pFGF21-289 mutant vector (Fig. 3E). Glucose also induced activation by 2-fold in the pFGF21-443 mutant vector, but the pFGF21-289 mutant vector did not respond to glucose (Fig. 3F). Interestingly, the basal human *FGF21* transcriptional activity was dramatically reduced in the pFGF21-443 deletion mutant vector as compared to the pFGF21-555 deletion mutant vector both in mouse primary hepatocytes and HepG2 cells (Fig. 3G, H). This reduction was regulated in a glucose-independent manner. These data indicate that the human *FGF21* promoter has at least one carbohydrate responsive region located at −443 to −289, and also an important region at −555 to −443 for basal transcriptional activity regulated in a glucose-independent manner.

### ChREBP binds to human FGF21 promoter regions

**Figure 4:**
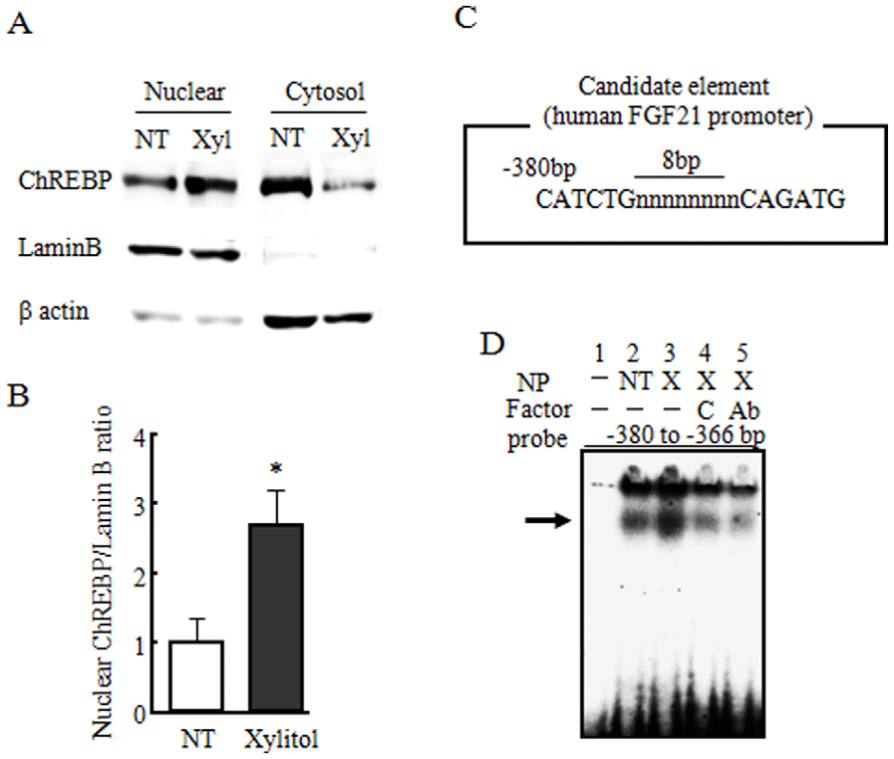
The human FGF21 promoter has a putative carbohydrate-responsive element. A, B: Changes in ChREBP protein abundance in cytosolic and nuclear fractions from mouse primary hepatocytes stimulated by 10 mM xylitol for 6 h. C: Putative carbohydrate responsive element (ChoRE) in the human *FGF21* promoter. D: An electrophoretic mobility shift assay was performed with the mouse primary hepatocyte nuclear fraction stimulated by xylitol (X) or not (NT). We used a putative ChoRE (−380 to −366 bp; lanes 1–5). Factor C is a competitor (*L-pk*; −142 to −124 bp) and Ab is ChREBP antibody. Arrow indicates DNA and protein complex. Data represent mean ± SE (n = 3). *: p<0.05 as compared to NT (non-treatment).

To investigate whether ChREBP can bind within the −443 to −289 bp region of the human *FGF21* promoter, we performed an electrophoretic mobility shift assay. Since the basal reporter activity was 15-fold higher in mouse primary hepatocytes than in HepG2 cells (Fig. 3B), we used mouse primary hepatocytes for the subsequent experiments. First, we confirmed that the nuclear extract from xylitol-stimulated mouse primary hepatocytes contained a larger amount of ChREBP protein as compared with control nuclear extract (Fig. 4A,B). We found that the human *FGF21* promoter contained a putative carbohydrate responsive element (ChoRE), composed of two imperfect E-boxes separated by an 8-bp gap, at position −380 to −366 bp (Fig. 4C). Xylitol-stimulated nuclear protein bound more strongly to the −380 to −366 bp region of the human *FGF21* promoter as compared with the control of non-stimulated nuclear protein (Fig. 4D). This binding was suppressed by preincubation with a competitor, the −146 to −124 bp region of *Lpk*, or ChREBP antibody (Fig. 4D). These results are consistent with the results of transcriptional activity evaluated by xylitol stimulation.

### ChREBP is needed to induce FGF21 gene transcription by glucose

**Figure 5:**
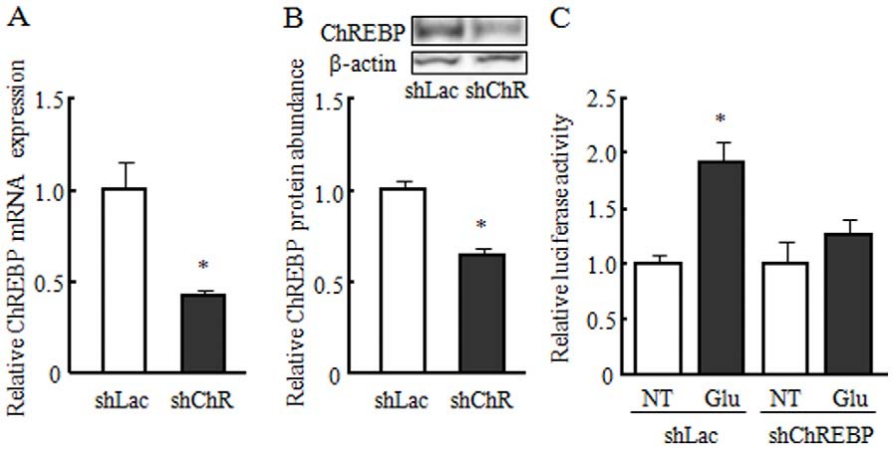
ChREBP is needed to induce FGF21 gene transcription by glucose. A, B: Changes in relative ChREBP gene expression and protein abundance at 48 h after transfection of either shLacZ or shChREBP vector in mouse primary hepatocytes. C: Changes in relative luciferase activity of the human −1.6 kb FGF21 reporter vector stimulated by 20 mM glucose 48 h after transfection of either shLacZ or shChREBP vector in mouse primary hepatocytes. The relative mRNA expression and protein abundance of ChREBP transfected with shLacZ vector was set as 1.0. The relative luciferase activity of NT (non-treatment) was 1.0. Data represent mean ± SE (n = 3). * p<0.05 as compared to shLacZ or NT.

To assess the effects of ChREBP on transcription of the human *FGF21* gene, we used a short hairpin RNA interference approach to inhibit ChREBP gene expression. After transfection of the pEnter-shChREBP vector, ChREBP gene expression and protein abundance were effectively reduced in mouse primary hepatocytes as compared to the control transfection (pEnter-shLacZ; Fig. 5A, B). Treatment with shChREBP diminished the glucose-induced upregulation of the human *FGF21* promoter activity observed in shLacZ-transfected cells (Fig. 5C). These results clearly show that ChREBP is needed to induce human *FGF21* gene expression by glucose stimulation.

## Discussion

**Figure 6:**
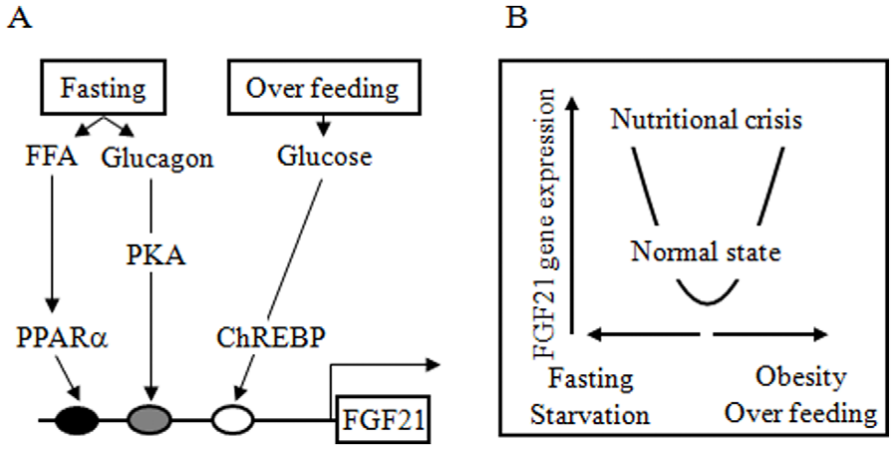
Schema summarizing the paradoxical regulation of human FGF21. A: Both fasting (PPARα and glucagon) and over feeding (glucose) signals activate human *FGF21* gene transcription. B: Human *FGF21* gene expression responds to nutritional crisis including fasting, starvation, over feeding and obesity.

In the present study, we examined the mechanisms regulating the human *FGF21* gene. We found that expression of the human *FGF21* gene is paradoxically regulated by both fasting and feeding signals (Fig. 6A). Two fasting signals, including PPARα and glucagon-PKA, increased expression of the human *FGF21* gene. Surprisingly, glucose and xylitol, which are feeding signals, also induced human *FGF21* gene expression through ChREBP activation. The human FGF21 promoter has at least one glucose-responsive region in the region −380 to −366 bp. In addition, the human FGF21 basal transcriptional activity was found to be independent of glucose, PPARα and glucagon-PKA.

It has been reported that fasting-induced liver *FGF21* gene expression is predominantly regulated by PPARα in mouse [4], [17]. PPARα knockout mice, however, can increase *FGF21* mRNA by fasting [4]. We previously showed that another fasting signal, glucagon, can also increase *FGF21* gene expression in rat primary hepatocytes [13]. Here, glucagon and forskolin, a PKA activator, significantly increased human *FGF21* promoter activities (Fig. 2C). This response was also seen in a PPRE deletion mutant vector, suggesting that human *FGF21* transcription is independently regulated by both PPARα and glucagon-PKA signaling. In the present study, we found that these fasting-induced mechanisms of *FGF21* regulation are conserved in the human *FGF21* promoter. We also showed that the human FGF21 promoter has one ChoRE in a different region from the PPRE. The human FGF21 promoter activities were dose-dependently increased by glucose or xylitol stimulation, as well as by a PPARα agonist (Fig. 2A, 3B and data not shown). Some human studies have reported that serum FGF21 concentrations are upregulated by fenofibrate [9]. In addition, as compared to normal glucose-tolerant subjects, plasma FGF21 levels were found to be higher in obese and IGT subjects [10]. These data suggest that hepatic *FGF21* mRNA expression is regulated, in part, through glucose, as well as in a PPARα-dependent manner, and this regulation can be reflected in serum FGF21 concentrations in humans.

Recently, Iizuka and colleagues reported that glucose can induce *FGF21* gene expression in rat primary hepatocytes [16]. They also showed that the mouse *FGF21* promoter has a putative ChoRE in the −74 to −52 bp region. In contrast, we identified a glucose-response region located at −380 to −366 bp in the human *FGF21* promoter. A typical ChoRE comprises two E-boxes separated by a 5-bp space, as observed in the mouse *FGF21* promoter [16]; however, in humans the ChoRE comprises two imperfect E-boxes, separated by an 8-bp space at −380 to −366. The space between the two E-boxes is important because a spacing of 4 bp between the CACGTG E-box motifs has been shown to result in a complete loss of induction [18]. By contrast, spacing of 6, 7 and 15 bp leads to a weak but significant response to glucose [18], indicating that the human FGF21 promoter has a weak ChoRE. In addition to this observation, we also found that the region from −555 to −443 bp of the human *FGF21* promoter region exerts an important role in activation of basic transcription. This region is separate from the PPRE, ChoRE and glucagon-PKA responsive elements.

In the present study, we showed that the human FGF21 transcriptional activity is regulated by both fasting and feeding signals. A recent study demonstrated that one-week prolonged fasting stimulation was required to increase FGF21 levels in humans [9]. Moreover, another human study reported that superphysiological levels of FFAs were required to induce FGF21 *in vivo* [19]. These observations suggest that FGF21 is upregulated by nutritional crisis. On the other hand, most human studies have linked obesity or IGT with increased FGF21 levels in humans [10], [11], because most humans in developed countries have more problems with over-nutrition than with starvation. These results indicate that the human FGF21 levels are increased in such abnormal physiological conditions. In fact, the responsivity of human FGF21 to glucose stimulation is weak, and the serum FGF21 levels of healthy subjects show no major changes related to feeding and fasting over the course of a day [9].

Our result suggests that FGF21 levels in human may be increased under malnutrition conditions, however Dostálová et al found that plasma FGF21 levels were lower in anorexic patients than control subject [20]. In contrast, Fazeli et al reported that FGF21 levels in anorexia nervosa patient were higher than those of control subjects after multiple adjustments including percent body fat [21]. They also mentioned that the balance of liver-derived FGF21 and adipose-derived FGF21 should be considered, because anorexic patients have extremely lower fat mass than healthy people. Additional studies are needed to elucidate the regulation and role of FGF21 in anorexia patients.

In addition, FGF21 play an important role in the adipocytes. FGF21 can stimulate glucose uptake and triglyceride degradation in the adipose tissue. FGF21 has been shown to be up-regulated during adipocyte differentiation [11]. ChREBP has also been shown to be up-regulated by adipocyte differentiation as well as glucose stimulation [22]. These inductions were also seen in PPARγ agonist stimulation. It seems that PPARγ and ChREBP can coordinately regulate FGF21 gene expression in adipocytes. However, additional studies are needed to investigate whether ChREBP directly and/or independently regulate FGF21 gene expression in the adipocytes to understand the role of FGF21 under over-nutrition or malnutrition observed in anorexic patients.

In conclusion, the human *FGF21* gene was paradoxically found to increase following both fasting and feeding signals in HepG2 and mouse primary hepatocytes. This unique dual regulation might be seen as paradoxical, and suggest that human FGF21 is increased with nutritional crisis, including starvation and overfeeding (Fig. 6B). Thus, human FGF21 levels may well be a useful marker to determine our nutritional status.
